# Improving the quality of HIV rapid testing in Ghana using the dried tube specimen-based proficiency testing program

**DOI:** 10.1371/journal.pone.0264105

**Published:** 2022-10-14

**Authors:** Bernard Nkrumah, Nnaemeka C. Iriemenam, Francis Frimpong, Mireille B. Kalou, Berenice Botchway, Rowland Adukpo, Keisha G. Jackson, Pawan Angra, Toni Whistler, Amitabh P. Adhikari, Stephen Ayisi-Addo, Michael A. Melchior

**Affiliations:** 1 Division of Global Health Protection, Center for Global Health, US Centers for Disease Control and Prevention, Accra, Ghana; 2 Division of Global HIV and TB, Center for Global Health, US Centers for Disease Control and Prevention, Abuja, Nigeria; 3 National AIDS/STI Control Program, Ghana Health Service, Accra, Ghana; 4 Division of Global HIV and TB, Center for Global Health, US Centers for Disease Control and Prevention, Port au Prince, Haiti; 5 Division of Global HIV and TB, Center for Global Health, US Centers for Disease Control and Prevention, Atlanta, Georgia, United States of America; 6 Division of Global Health Protection, Center for Global Health, US Centers for Disease Control and Prevention, Atlanta, Georgia, United States of America; 7 Division of Global HIV and TB, Center for Global Health, US Centers for Disease Control and Prevention, Harare, Zimbabwe; University of Washington, UNITED STATES

## Abstract

**Background:**

The introduction of human immunodeficiency virus (HIV) antibody rapid testing (RT) in resource-limited settings has proven to be a successful intervention to increase access to prevention measures and improve timely linkage to care. However, the quality of testing has not always kept pace with the scale-up of this testing strategy. To monitor the accuracy of HIV RT test results, a national proficiency testing (PT) program was rolled out at selected testing sites in Ghana using the dried tube specimen (DTS) approach.

**Methods:**

Between 2015 and 2018, 635 HIV testing sites, located in five regions and supported by the U.S. President’s Emergency Plan for AIDS Relief (PEPFAR), were enrolled in the HIV PT program of the Ghana Health Service National AIDS/STI Control Programme. These sites offered various services: HIV Testing and Counselling (HTC), prevention of mother-to-child transmission (PMTCT) and Antiretroviral Treatment (ART). The PT panels, composed of six DTS, were prepared by two regional laboratories, using fully characterized plasma obtained from the regional blood banks and distributed to the testing sites. The results were scored by the PT providers according to the predefined acceptable performance criteria which was set at ≥ 95%.

**Results:**

Seven rounds of PT panels were completed successfully over three years. The number of sites enrolled increased from 205 in round 1 (June 2015) to 635 in round 7 (December 2018), with a noticeable increase in Greater Accra and Eastern regions. The average participation rates of enrolled sites ranged from 88.0% to 98.0% across the PT rounds. By round 7, HTC (257/635 (40.5%)) and PMTCT (237/635 (37.3%)) had a larger number of sites that participated in the PT program than laboratory (106/635 (16.7%)) and ART (12/635 (1.9%)) sites. The average testing performance rate improved significantly from 27% in round 1 to 80% in round 7 (p < 0.001). The highest performance rate was observed for ART (100%), HTC (92%), ANC/PMTCT (90%) and Laboratory (89%) in round 5.

**Conclusion:**

The DTS PT program showed a significant increase in the participation and performance rates during this period. Sub-optimal performances observed was attributed to non-compliance to the national testing algorithm and testing technique. However, the implementation of review meetings, peer-initiated corrective action, supportive supervisory training, and mentorship proved impactful. The decentralized approach to preparing the PT panels ensured ownership by the region and districts.

## Background

HIV testing is the entry point to care and treatment and a vital component for avoiding onward transmission [1]. Over the past decade, HIV testing services have increased significantly, with more than two thirds of all people living with HIV globally knowing their status in 2016 [2]. The introduction of HIV antibody rapid testing is a proven intervention to ensure timely linkage to care [3] and same-day initiation of antiretroviral therapy (ART) for HIV-infected individuals in sub-Saharan Africa [4]. In Ghana, the number of individuals tested increased from over 1 million in 2016 to 1.8 million in 2020 [5].

Despite the increase in HIV testing volumes, in 2015 concerns were raised about the sub-optimal quality of HIV testing. This was largely attributed to: assay limitations, deviation from testing algorithms and procedures, under-trained and over-worked staff, substandard storage conditions of reagents and test kits, use of inappropriate testing strategies, and sub-optimal national testing algorithms [6]. On average, 0.4% and 3.1% of diagnoses conducted in Swaziland among adults were false negatives or false positives respectively [7]. Previous studies reported that up to 6.6% of HIV patients who initiated ART had been misdiagnosed [6]. In Ghana, 5.8% of pregnant women received false negative HIV test results [8]. A false-negative HIV diagnosis could exempt an infected patient from being linked to prevention, treatment, and care services [9]. False negative test results in a person means they can continue infecting other people hence spreading the disease. A false-positive HIV diagnosis is likely to result in a patient being wrongfully initiated on life-long ART, increased potential for being stigmatized and discriminated against [10], and negative impact on community and family relationships [9]. Testing quality issues and misdiagnoses undermine the goal of HIV testing services and make it difficult to reach the UNAIDS 95-95-95 goal by 2030 [11].

The implementation of quality assurance (QA) measures effectively improve the confidence in the testing process by monitoring the outcomes through measures that minimize the variability and increase the accuracy of results interpretation [12]. In 2015, the World Health Organization (WHO) released guidelines on consolidated HIV testing services recommending the implementation of comprehensive QA programs which includes proficiency testing (PT) programs [13]. PT consists of sending blinded samples to testing sites where they are tested as part of the routine samples and the results returned to the PT providers for analysis and inter-laboratory comparison. By design, PT programs aim to assess the performance of individual laboratories and to verify the accuracy of the test results. These measures have been documented to improve the quality of testing for various diseases and analytes [14]. Most PT panels require transportation in cold temperatures (2–8°C) to maintain the integrity of the specimens. This has limited the ability of countries to participate and expand PT programs to remote locations in resource-limited settings (RLS) [15]. To address this logistical challenge, dried tube specimen (DTS), an alternative specimen type for PT and quality control, was developed to allow for implementation of quality monitoring of HIV testing at sites in RLS [16] and has since been expanded to include other diseases and biomarkers [15, 17–19].

In Ghana, the HIV PT program started in 2008 in 40 laboratories using plasma specimens to monitor the laboratories’ performance in conducting HIV rapid testing. However, maintaining cold chain during transportation limited a full-scale implementation. With the adoption of the WHO *Treat All* recommendations [20] in 2016, the country recognized the need to expand the PT program to all testing points to ensure the accuracy of results provided to individuals and the initiation of ART. Between June 2015 and December 2018, Ghana modified the plasma specimen-based HIV PT program by adopting DTS as an alternative specimen type. This report describes the HIV rapid testing performance at the enrolled testing sites.

## Methods

### Site selection and enrollment

The study sites were selected from five regions (Greater Accra, Eastern, Western, Ashanti, and Brong Ahafo), supported by the U.S. President’s Emergency Plan for AIDS Relief (PEPFAR), with demonstrated high yield of HIV positive results. Selected sites were enrolled in the PT program in a phased approach starting with 205 pilot sites from the Greater Accra and Eastern regions in June 2015 in round 1. The program was gradually expanded to additional testing sites in Greater Accra and Eastern regions and to the Western, Ashanti, and Brong Ahafo regions, bringing the total number of participating sites to 635 by the end of December 2018. At enrollment, the sites were assigned a unique PT code. In accordance with the national HIV testing algorithm, standard operating procedures (SOPs) were developed on DTS panel reconstitution, and documentation and reporting of the PT results. All staff performing the HIV testing were trained on these SOPs.

### Preparation of HIV DTS panels

HIV DTS PT panels were prepared as previously described [16], from pooled plasma samples obtained from de-identified rejected blood units collected from various regional blood transfusion centers [16]. The pooled plasma specimens were subsequently characterized and validated using the national HIV serial testing algorithm which includes First Response 1-2-0 Test Kit (Premier Medical Corporation Ltd., Kachigam, India), Oraquick Rapid HIV-1 Antibody Test (OraSure Technologies, Inc, Bethlehem, PA) and INNO-LIA HIV I/II Score test (Fujirebio, Ghent, Belgium) [21]. For each site distribution, the PT panel was comprised of a total of six DTS specimens. Each panel specimen was assigned a unique identifier, corresponding to the HIV sero-status. This information was only known to the regional and national teams coordinating the PT program. The panels were stored and shipped at room temperature to the sites.

### Quality assurance testing of PT panels

To ensure the highest quality standards, two regional laboratories (Eastern Regional Hospital Laboratory in Eastern Region and Sekondi Public Health laboratory in Western Region) that were involved in the Strengthen Laboratory Management Toward Accreditation (SLMTA) program and had been assessed to have at least a three stars rating using the WHO Stepwise Laboratory Quality Improvement Process Towards Accreditation (SLIPTA) checklist [22] prepared the PT panels. For each round, a quality check was conducted to ensure the integrity of the panels during the storage. Approximately 10% of panels were randomly selected and retested by the national quality assurance team using the national HIV testing algorithm, prior to their distribution. The quality check results were compared to the initial results, and any specimen with a discrepant result was replaced and not included in the distributed PT panel.

### Packaging and transportation of the PT materials

For each panel distribution, six DTS tubes were packaged along with one tube of reconstitution buffer (Phosphate Buffered Saline with Tween 20 (PBS-T)), one 0.3 ml disposable Pasteur pipette, a result reporting form and a testing instructions sheet. The PT packages were delivered twice a year to the testing sites using either postal services, a local courier, or hand delivery by the district HIV focal persons.

### PT sample testing procedure

Upon receipt, the testing staff inspected the package for integrity before reconstituting the DTS tubes by adding 7 drops (approximately 200μl) of the PBS-T to each specimen tube, using the provided 0.3 ml disposable Pasteur pipette. The tubes were tightly closed, mixed by gentle inversion, and the DTS pellets were allowed to dissolve at room temperature for 2 to 4 hours. The reconstituted DTS were then tested using the national HIV rapid testing algorithm [21]. Test results were recorded on the report form and submitted to the respective district or regional focal persons or directly to the national team within two weeks after the panels’ receipt.

### PT data scoring

The regional focal persons or the national PT program administrator were responsible for entering the test results into the electronic PT (ePT) data management tool. The data was reviewed and analyzed using a two-step scoring criteria: 1) panel test results. Each panel specimen identified correctly received 15 points, for a maximum of 90 points and 2) documentation (10 points). Several quality indicators were monitored on the result form: 1) panel receipt date, 2) sample rehydration date, 3) testing date and time, 4) test kit information (e.g., type of test kits used, test kit expiration date and the lot number), and 5) supervisor signature indicating the review of the results form before their submission. Each of these elements received 2 points, if they were accurately documented. A site performance score ≥95 points was considered satisfactory performance. Performance rate was defined as the proportion panels correctly tested and reported, and expreesed as a percentage of the individual site scores. Participation rate however, was defined as the number of testing sites that received and submitted their test results before the deadline.

### Statistical analysis

Univariable analysis were based on Pearson χ^2^ test for the comparison of proportions. Descriptive analyses of testing sites by site type, participation and performance rates were done for each round. All statistical analyses used were two-tailed tests and p ≤ 0.05 was considered as statistically significant. Data analysis was conducted using IBM SPSS Statistics version 21.0 (IBM Corporation, Armonk, NY, USA).

### Ethical considerations

The study was reviewed by the Institutional Review Board of the Center for Global Health (CGH) Office of the Associate Director of Science (ADS) in accordance with US Centers for Disease Control and Prevention (CDC) human research protection procedures and was determined to be a non-human subject research with a Tracking number: 2016–195. Ethical approval was waived. Verbal consent was obtained from all enrolled sites and documented as part of study reports.

## Results

Between 2015 and 2018, seven rounds of DTS based PT were completed. In total, 635 testing sites representing four testing site types, were enrolled in the program. These included 272 (42.8%) HIV HTC sites, 244 (38.4%) ANC or PMTCT sites, 107 (16.9%) laboratories and 12 (1.9%) ART sites, by the end of 2018.

**Fig 1:**
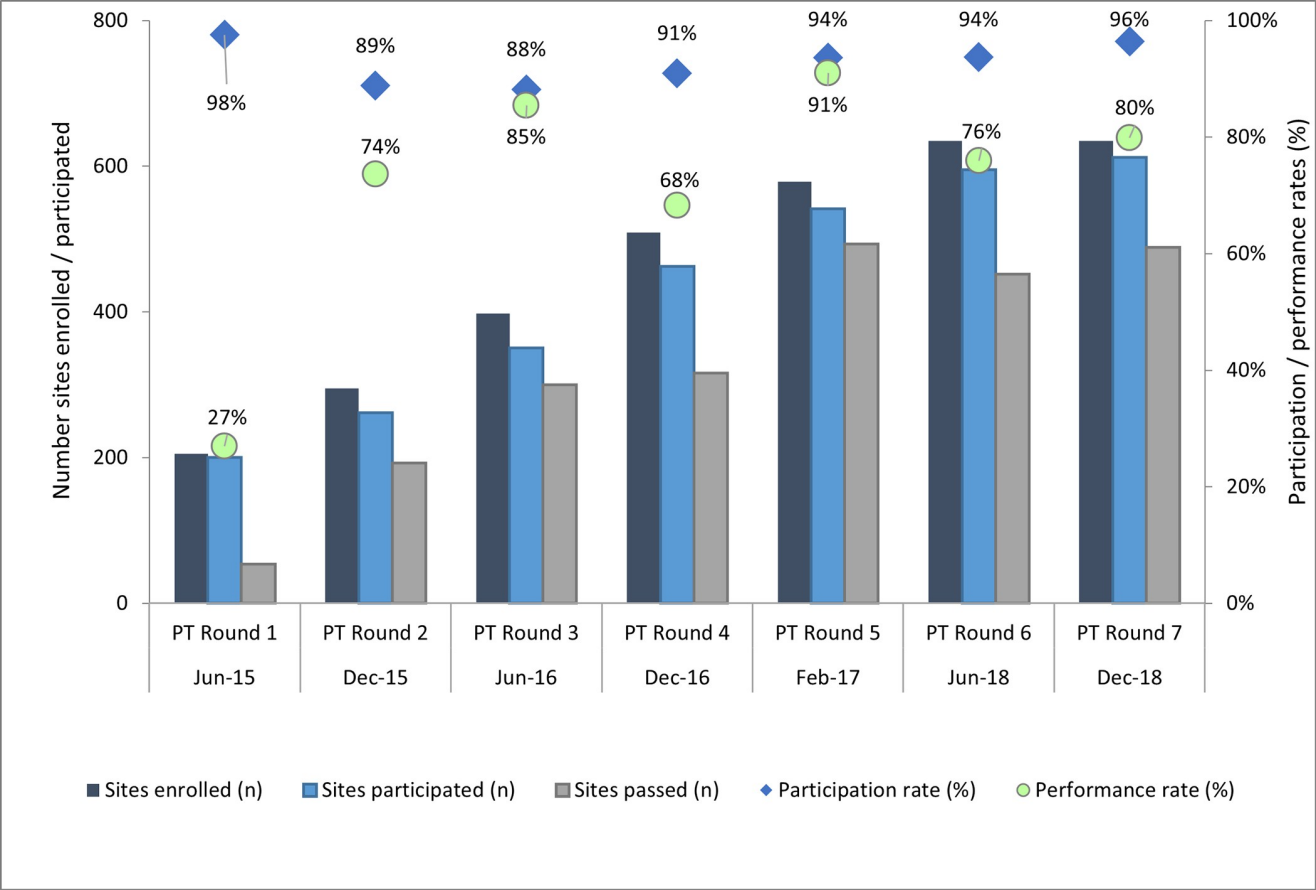
Overall study statistics of HIV testing sites from round 1–7. Trends of HIV testing sites enrolled (N = 635) and participated in the proficiency testing (PT) program, their participation and performance rates between June 2015 and December 2018 (PT rounds 1 to 7) in 5 regions in Ghana.

**Fig 2:**
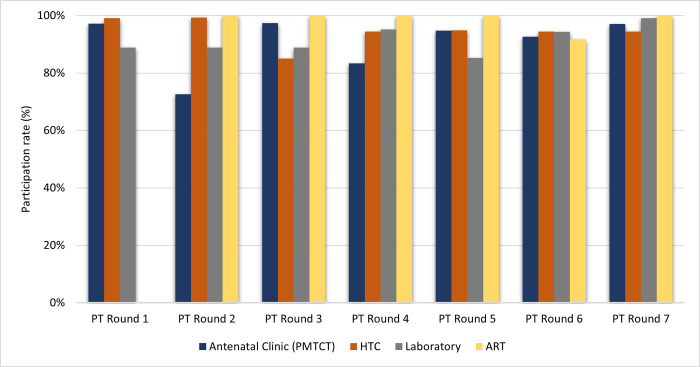
Participation rate by type of testing facility from round 1–7. High participation rates were observed among all testing sites. HTC and ART testing sites maintained consistent participation rates throughout the period of the study whilst Laboratory and PMTCT testing sites performance rates fluctuated within the same period.

The average participation rates of enrolled sites ranged from 88.0% to 98.0%, with no significant statistical difference across rounds (p = 0.159). These rates decreased from 98.0% (89–99%) in round 1 to 88.0% (85–100%) in round 3 and increased gradually to reach 96.0% (94–100%) during round 7 (Fig 1). The highest average participation rates were observed among HTC 94.4% (85–99%), laboratory 91.4% (85–99%) and ANC or PMTCT 90.7% (73–97%) sites (Fig 2).

Although there was a substantial reduction in the proportion of ANC or PMTCT sites that submitted their results in the 2^nd^ round (69/95 (72.6%)), their participation rate remained relatively high (97.0%) throughout the subsequent rounds. Similarly, all twelve ART clinics enrolled in the program during the 2^nd^ round, returned their results for each PT panel distribution, except in round 6, where the participation rate dropped from 100% to 92.0% (Fig 2).

The average regional participation rates varied between 61.0% and 98.0% across all seven rounds. The highest number of testing facilities participating in the program were in Eastern 97.3% (96%-100%), Greater Accra 96.4% (90%-99%), and Western 95.8% (91%-100%) regions. Participation rates were lowest for Brong Ahafo (18.0% (17.0–99.0%)) and Ashanti (37.0% (37.0–95.0%)) regions but these improved during the program (Table 1).

**Table 1:** Trends of the HIV sites proficiency testing participation and performance across testing sites over seven PT rounds.

Test Modality	PT Round 1			PT Round 2			PT Round 3			PT Round 4			PT Round 5			PT Round 6			PT Round 7		
	N	PAT (%)	PET (%)	N	PAT (%)	PET (%)	N	PAT (%)	PET (%)	N	PAT (%)	PET (%)	N	PAT (%)	PET (%)	N	PAT (%)	PET (%)	N	PAT (%)	PET (%)
ANC/PMTCT	71	97	17	95	73	86	77	97	88	169	83	68	228	95	90	244	93	69	244	97	73
HTC	116	99	29	145	99	74	255	85	85	252	94	66	272	95	92	273	95	81	272	94	88
Laboratory	18	89	56	54	89	56	63	89	84	84	95	78	75	85	89	106	94	79	107	99	77
ART	-	-	-	1	100	-	3	100	100	4	100	25	4	100	100	12	92	91	12	100	83
**Overall**	**205**	**95**	**34**	**295**	**87**	**72**	**398**	**90**	**86**	**509**	**91**	**71**	**579**	**92**	**90**	**635**	**94**	**76**	**635**	**97**	**79**

PAT: Participation Rate PET: Performance Rate.

The average performance rates among testing sites that participated at the start of the program increased significantly from 27.0% in PT round 1 to 91.0% in PT round 5 (P < 0.001), however with a noticeable 80.0% decrease in round 7 (Fig 1). Although the site performance rates improved over time, it was inconsistent from round to round, with the highest performance rate observed in round 5 (Fig 1), following a series of supportive supervisory visits to the testing sites. Average performance by laboratory sites remained low and varied between 56.0% in round 1 and 89.0% in round 5 (Table 1). The performance rates of the sites located in Eastern, Greater Accra and Western regions were consistently high after the first round (Table 2). In the Ashanti region however, the performance rates were inconsistent and fluctuated between 50.0% and 89.0% (Table 2).

**Table 2:** Trends of the HIV sites proficiency testing participation and performance across geographical regions over seven PT rounds.

Regions	PT Round 1			PT Round 2			PT Round 3			PT Round 4			PT Round 5			PT Round 6			PT Round 7		
	N	PAT (%)	PET (%)	N	PAT (%)	PET (%)	N	PAT (%)	PET (%)	N	PAT (%)	PET (%)	N	PAT (%)	PET (%)	N	PAT (%)	PET (%)	N	PAT (%)	PET (%)
Eastern Region	103	96	13	109	100	90	113	96	90	115	97	91	115	97	97	128	97	83	128	98	94
Greater Accra Region	102	99	41	111	99	64	118	90	84	112	97	97	111	97	98	122	97	85	122	96	95
Western Region	-	-	-	12	100	40	119	91	29	112	96	49	112	97	81	123	96	45	123	95	51
Ashanti Region	-	-	-	12	83	50	19	37	80	111	95	67	112	78	89	121	79	77	121	95	77
Brong Ahafo Region	-	-	-	33	18	75	10	50	86	36	17	33	106	99	91	118	98	84	118	98	82
Others*	-	-	-	18	83	60	19	89	82	23	91	71	23	91	76	23	100	78	23	96	77
**Total**	**205**	**98**	**27**	**295**	**67**	**53**	**398**	**61**	**62**	**509**	**67**	**56**	**579**	**78**	**76**	**635**	**78**	**62**	**635**	**80**	**67**

Round: Distribution of the proficiency testing panel to the sites. Each round the number of sites enrolled and/or participating may vary.

N: Number of sites enrolled in the proficiency testing program that participated.

Performance rate: Proportion of sites enrolled and participating in the program with a satisfactory score divided by the total number of sites enrolled and participating in the program

Others* (Central Region, Northern Region, Upper East Region, Upper West Region and Volta Region).

PAT: Participation Rate. PET: Performance Rate.

**Fig 3:**
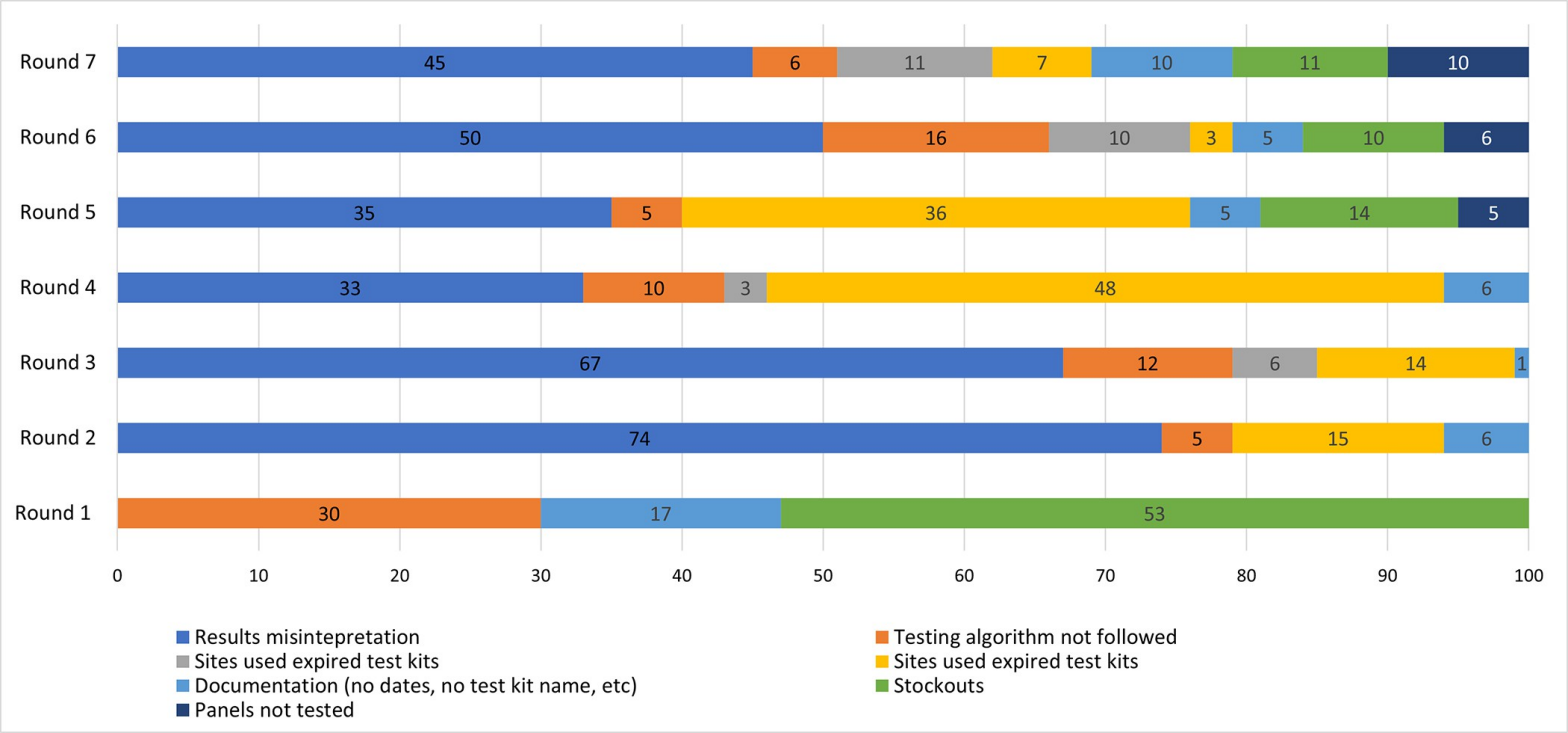
Reasons for unsatisfactory performance by testing sites. Unsatisfactory performance during the study period was attributed to a number of reasons with results misinterpretation being the highest. There was a major stockout of HIV tests kits in round 1 and minor ones recorded in rounds 5, 6 and 7. Testers not following the national HIV testing algorithm also attributed to non-performance throughout the study.

The proportion of sites that did not follow the national HIV rapid testing algorithm decreased significantly from 30.0% in first round to 2.0% in round 5, however a slight increase was observed at round 7 (6.0%). Other reasons for unsatisfactory results included: 1) improper documentation of the test kit information such as name of test used, expiration date, lot number; 2) test performed without using the Oraquick Rapid HIV-1 Antibody test to confirm all specimens initially reactive by the First Response HIV 1-2-0 Card test because of stock-outs, which reduced by round 7 (Fig 3).

## Discussion

With the rapid expansion of the HIV testing services in Ghana to improve linkage into care, it is critical to ensure only HIV confirmed positive individuals are initiated on treatment. Between June 2015 and December 2018, seven PT rounds were completed successfully. There was a high level of testing site participation and submission of test results across all rounds. Overall, the increase in the participation rates was attributed to the regionalization of the program, with significant involvement of the regional administrative teams that managed the program at the regional and district levels. These teams enrolled all eligible testing sites into the program, distributed the PT panels to sites, and ensured test results were submitted by due dates. For testing sites in remote locations, test results were submitted via WhatsApp Messenger accounts created by the regional teams specifically for that purpose. The use of the WhatsApp Messenger application has been shown to be an effective communication means between health care professionals [23] or between health care professionals and the general public [24].

Sites performance rates at the initiation of the program were low compared to other countries [25]. The sub-optimal performance rates observed among many sites were primarily due to a deviation from the national testing algorithm. Although most test providers appeared to be familiar with the serial testing algorithm adopted by Ghana a few years prior to the DTS PT program’s roll-out, it was evident that they did not understand the basic principle of the serial testing algorithm. Non-compliance with the algorithm could be attributed to: 1) limited and inconsistent provider training, 2) high staff attrition, or 3) many trained providers who had retired without sufficient knowledge transfer to new providers offering HIV testing services. Furthermore, the consistent shortage of Oraquick test kits was a contributing factor to the lack of algorithm compliance. This also points towards a bigger challenge of limited national and regional level monitoring to enforce proper usage and accountability of test kits. These observations were corroborated by a previous study conducted in South Africa, in which only 43% of providers used a tie-breaker test to confirm discordant results between screening and confirmatory tests, as recommended by the national algorithm. These testers indicated that they confirmed the test results if requested by a supervisor, when the test kits were available, or because they were trained [26]. Surprisingly, a recent evaluation conducted in South Africa showed that although the PT performance rates were high (>95%), more than 76% of the facilities continued to use the previous testing algorithm despite the change that occurred. The facilities reported following the recommendation to use the test kits from the previous algorithm because of the overstock of test kits, and less than 0.3% of them actually deviated from the previous algorithm [27].

Performance rates varied among the different testing types throughout the study period, suggesting that initial trainings needed to be better tailored to the target audiences and settings. This is supported by findings from a recent review study in Haiti which showed that PT performance was better when testing was performed in a laboratory setting by laboratory scientists than when conducted in non-laboratory setting by other professionals at the different testing types [28]. Further, the importance of training and supportive supervision was highlighted by the conclusion of a study conducted in Zambia, which showed an improvement in HIV testing accuracy among all cadres after the provision of targeted training and supervisory visits [29].

Because previous supervisory visits to HIV testing sites reported inconsistent use of testing registers, the PT program included the review and scoring of key indicators that assessed the completeness and accuracy of documentation to reinforce this quality practice. Interestingly, either incomplete or the lack of documentation of the final test results or test kits information were noted as some of the reasons for unsatisfactory PT performance across PT rounds. As observed in Ghana, a survey conducted in Tanzania to determine the level of implementation of key quality domains revealed that 50% of the facilities assessed used the testing registers and for those that did not, they considered that completing the registers was additional work [30]. In South Africa however, although the testing registers were used by the facilities assessed, 46% of them did not document invalid test results either because they never had an invalid result or those results were recorded incorrectly [27].

A comprehensive review of the initial testing performance by the regional teams resulted in several observations. First, the large centralized training organized in the Greater Accra and Eastern regions were sub-optimal. The large number of participants (100 and above) in the session prevented direct interactions between the facilitators and participants and the practical sessions and supervision during group work sessions were ineffective. Secondly, the training content did not fully address the basic testing principles, did not include adequate number of practical sessions, did not account for language barrier (including local dialects) to explain complex concepts and terminologies, and finally did not include sufficient exercises on documentation of test results. Thirdly, some of the participants invited to the training were not routinely involved in the performance of HIV testing, hence they had limited knowledge and skills in the different test procedures. These observations supported previous study findings suggesting that although HIV RT is simple and easy to perform, test providers’ insufficient training and inexperience are a limitation to achieving accurate diagnostic testing [31]. Hence, as the country scaled up the program, these lessons were taken into account to improve follow-up training design by:1) decentralizing training to the district-level, allowing for smaller training size and increased time for facilitator-participant interactions, 2) the theoretical contents and sessions were improve with diagrams, pictures and infographics, 3) the number of practical sessions was increased to incorporate more activities, including exercises on documentation of test results and 4) the language and terminology used were simplified and localized for better understanding.

The significant decline in performance rates in round 4 (Table 1), across the different testing sites, types and regions, prompted the implementation of remedial measures to help identify shortcomings and training needs, and improve overall quality processes at the sites. The measures included regular supportive supervision visits and coordinated monitoring activities that involved regional administrative teams, district laboratory managers, HIV coordinators and national level technical staff. Additionally, peer-to-peer support was provided at the district level by the high performing sites to sites with sub-optimal performance; the sites assisted with the implementation of the corrective actions. The combination of these interventions have been shown to be effective as site performance improved overtime [32, 33].

### Lessons learned

First, the introduction of electronic options (e.g., emails and WhatsApp) to submit the PT results were considered by the testing sites as valuable and reliable alternatives. Secondly, the review meetings held at the end of each round and after testing results were analyzed helped the regional and national teams to discuss performance and develop corrective action and training plans for each site before the next round. This intervention ensured each round was adequately addressed and allowed for the development of round-specific corrective actions and implementation plans. Lastly, in most cases where the PT results were not reviewed and/or approved prior to their submission, mistakes and omissions were very common on the result forms and they affected the site performance. Thus, this step was critical to ensure that the PT forms were completed accurately.

## Conclusion

Using DTS as an alternative proficiency testing specimen has allowed the Ghana Health Service National AIDS/STI Control Programme to expand the PT program to remote locations and all testing types. The decentralization of the PT panels’ preparation was quite innovative. Most PT programs are generally managed by a national entity. Decentralization has ensured that the program is implemented and owned by the regions and districts with support from the national level. Root cause analyses of the national algorithm’s deviation revealed the need to rethink the training model, strategy and implementation of interventions such as review meetings, peer initiated remedial action training, supportive supervision for both the administrative teams and testers and test kits usage monitoring and accountability. As the country strives to achieve the UNAIDS targets by 2030, it is important to adopt and rapidly scale up comprehensive quality programs such as the HIV rapid testing continuous quality improvement initiative, as the PT program alone is a limited quality assurance activity and does not address other quality management domains. A careful analysis of PT findings has the potential to unearth the problems hidden in the plain sight and addressing them may help make the Ghana HIV program robust, reliable, and resilient.

## References

[1] Staveteig S, Croft TN, Kampa KT, Head SK. Reaching the ‘first 90’: Gaps in coverage of HIV testing among people living with HIV in 16 African countries. PloS One. 2017;12(10):e0186316. doi: 10.1371/journal.pone.0186316 29023510 PMC5638499

[2] UNAIDS. Ending AIDS: progress towards the 90–90–90 targets. Geneva: Joint United Nations Programme on HIV. AIDS. 2017.

[3] Smith A, Sabidó M, Camey E, Batres A, Casabona J. Lessons learned from integrating simultaneous triple point‐of‐care screening for syphilis, hepatitis B, and HIV in prenatal services through rural outreach teams in Guatemala. International Journal of Gynecology & Obstetrics. 2015;130:S70–S2. doi: 10.1016/j.ijgo.2015.04.009 25968489

[4] Heller T, Kunthea S, Bunthoeun E, Sok K, Seuth C, Killam W, et al. Point-of-care HIV testing at antenatal care and maternity sites: experience in Battambang Province, Cambodia. International Journal of STD & AIDS. 2011;22(12):742–7. doi: 10.1258/ijsa.2011.011262 22174058

[5] National AIDS/STI Control Program. HIV Service Data: Jan-Dec 2020. 2021.

[6] Johnson CC, Fonner V, Sands A, Ford N, Obermeyer CM, Tsui S, et al. To err is human, to correct is public health: a systematic review examining poor quality testing and misdiagnosis of HIV status. Journal of the International AIDS Society. 2017;20:21755. doi: 10.7448/IAS.20.7.21755 28872271 PMC5625583

[7] Khan S, Mafara E, Pasipamire M, Spiegelman D, Mazibuko S, Ntshalintshali N, et al. Identification of misdiagnosed HIV clients in an Early Access to ART for All implementation study in Swaziland. Journal of the International AIDS Society. 2017;20:21756. doi: 10.7448/IAS.20.7.21756 28872273 PMC5625592

[8] Tetteh AK, Agyarko E. Discordant HIV Test Results: Implications on Perinatal and Haemotransfusion Screening for HIV Infection, Cape Coast, Ghana. Journal of Sexually Transmitted Diseases. 2017;2017. doi: 10.1155/2017/2857397 29119035 PMC5651149

[9] Johnson C, Fonner V, Sands A, Tsui S, Ford N, Wong V, et al. ANNEX 14. A report on the misdiagnosis of HIV status. WHO/HIV/2015.33. 2015.

[10] Kontomanolis EN, Michalopoulos S, Gkasdaris G, Fasoulakis Z. The social stigma of HIV–AIDS: society’s role. HIV AIDS (Auckl). 2017;9:111. doi: 10.2147/HIV.S129992 28694709 PMC5490433

[11] UNAIDS. Fast-Track: ending the AIDS epidemic by 2030. 2014.

[12] Martin R, Hearn TL, Ridderhof JC, Demby A. Implementation of a quality systems approach for laboratory practice in resource-constrained countries. AIDS. 2005;19:S59–S65. doi: 10.1097/01.aids.0000172878.20628.a8 15930842

[13] WHO. Consolidated guidelines on HIV testing services. 2015. 42766700

[14] Drammeh B, Laperche S, Hilton JF, Kaidarova Z, Ozeryansky L, De A, et al. Proficiency Testing of Viral Marker Screening in African Blood Centers—Seven African Countries, 2017. Morbidity and Mortality Weekly Report. 2019;68(42):947. doi: 10.15585/mmwr.mm6842a3 31652252 PMC6812837

[15] Zakaryan A, Manjengwa J, Danielyan H, Tumanyan P, Davtyan Z, Kachuwaire O, et al. Dried tube specimen preparation and stability validation for brucellosis serological external quality assessment and quality control materials in resource-limited settings. Authorea. 2020. doi: 10.22541/au.159285443.39521462

[16] Parekh BS, Anyanwu J, Patel H, Downer M, Kalou M, Gichimu C, et al. Dried tube specimens: a simple and cost-effective method for preparation of HIV proficiency testing panels and quality control materials for use in resource-limited settings. Journal of Virological Methods. 2010;163(2):295–300. doi: 10.1016/j.jviromet.2009.10.013 19878697

[17] Nguyen S, Ramos A, Chang J, Li B, Shanmugam V, Boeras D, et al. Monitoring the quality of HIV-1 viral load testing through a proficiency testing program using dried tube specimens in resource-limited settings. Journal of Clinical Microbiology. 2015;53(4):1129–36. doi: 10.1128/JCM.02780-14 25609733 PMC4365191

[18] Dorkenoo AM, Kouassi KC, Koura AK, Adams ML, Gbada K, Katawa G, et al. The use of dried tube specimens of Plasmodium falciparum in an external quality assessment programme to evaluate health worker performance for malaria rapid diagnostic testing in healthcare centres in Togo. Malaria Journal. 2021;20(1):1–10. 33472640 10.1186/s12936-020-03569-y PMC7819240

[19] Benzaken AS, Bazzo ML, Galban E, Pinto ICP, Nogueira CL, Golfetto L, et al. External quality assurance with dried tube specimens (DTS) for point-of-care syphilis and HIV tests: experience in an indigenous populations screening programme in the Brazilian Amazon. Sexually Transmitted Infections. 2014;90(1):14–8. doi: 10.1136/sextrans-2013-051181 24031029

[20] WHO. Consolidated guidelines on the use of antiretroviral drugs for treating and preventing HIV infection: Recommendations for a public health approach—Second edition. 2016. 27466667

[21] Ghana Health Service. PMTCT Handbook for Healthcare Providers in Ghana. 2014.

[22] Nkrumah B, van der Puije B, Bekoe V, Adukpo R, Kotey NA, Yao K, et al. Building local human resources to implement SLMTA with limited donor funding: The Ghana experience. African Journal of Laboratory Medicine. 2014;3(2). doi: 10.4102/ajlm.v3i2.214 26937417 PMC4770820

[23] Pimmer C, Mhango S, Mzumara A, Mbvundula F. Mobile instant messaging for rural community health workers: a case from Malawi. Global Health Action. 2017;10(1):1368236. doi: 10.1080/16549716.2017.1368236 28914165 PMC5645652

[24] Giordano V, Koch H, Godoy-Santos A, Belangero WD, Pires RES, Labronici P. WhatsApp messenger as an adjunctive tool for telemedicine: an overview. Interactive Journal of Medical Research. 2017;6(2):e6214. doi: 10.2196/ijmr.6214 28733273 PMC5544893

[25] Muchiri SM, Osman S, Night D, Muia E, Kaiguri P, Kimotho J, et al. Performance of selected HIV testing centers in a HIV Proficiency Testing Scheme in Kenya: a case study. African Journal of Pharmacology and Therapeutics. 2016;5(1).

[26] Mwisongo A, Peltzer K, Mohlabane N, Tutshana B. The quality of rapid HIV testing in South Africa: an assessment of testers’ compliance. African Health Sciences. 2016;16(3):646–54. doi: 10.4314/ahs.v16i3.2 27917195 PMC5111989

[27] Woldesenbet SA, Kalou M, Mhlongo D, Kufa T, Makhanya M, Adelekan A, et al. An overview of the quality assurance programme for HIV rapid testing in South Africa: Outcome of a 2-year phased implementation of quality assurance program. PloS One. 2019;14(9):e0221906. doi: 10.1371/journal.pone.0221906 31557176 PMC6762059

[28] Louis FJ, Excellent ML, Anselme R, Buteau J, Stanislas M, Boncy J, et al. External quality assessment for HIV rapid tests: challenges and opportunities in Haiti. BMJ Global Health. 2018;3(6). 10.1136/bmjgh-2018-001074 PMC6254742 30498590

[29] Mwangala S, Musonda KG, Monze M, Musukwa KK, Fylkesnes K. Accuracy in HIV rapid testing among laboratory and non-laboratory personnel in Zambia: observations from the national HIV proficiency testing system. PloS One. 2016;11(1):e0146700. doi: 10.1371/journal.pone.0146700 26745508 PMC4706302

[30] Mashauri F, Siza J, Temu M, Mngara J, Kishamawe C, Changalucha J. Assessment of quality assurance in HIV testing in health facilities in Lake Victoria zone, Tanzania. Tanzania Journal of Health Research. 2007;9(2):110–4. 10.4314/thrb.v9i2.14312 17722413

[31] Chiu Y-HC, Ong J, Walker S, Kumalawati J, Gartinah T, McPhee DA, et al. Photographed rapid HIV test results pilot novel quality assessment and training schemes. PLoS One. 2011;6(3):e18294. 21483842 10.1371/journal.pone.0018294 PMC3069085

[32] Learmonth KM, McPhee DA, Jardine DK, Walker SK, Aye T-T, Dax EM. Assessing proficiency of interpretation of rapid human immunodeficiency virus assays in nonlaboratory settings: ensuring quality of testing. Journal of Cinical Microbiology. 2008;46(5):1692–7. doi: 10.1128/JCM.01761-07 18353938 PMC2395071

[33] Mosoke P, Ndasi J, Dimite L, Efuetakoa C, Etogo B, Jackson K, et al. Innovative Approach to Improving Adherence to Quality Standards at HIV Rapid Testing Sites in Cameroon using the Stepwise Process for Improving the Quality of HIV Rapid Testing (SPI-RT) Checklist. International Journal of Trend in Scientific Research and Development. 2020;4.

